# Percutaneous Coronary Intervention Is Not Superior to Optimal Medical Therapy in Chronic Coronary Syndrome: A Meta-Analysis

**DOI:** 10.3390/jcm12041395

**Published:** 2023-02-09

**Authors:** Ibadete Bytyçi, Defrim Morina, Sefer Bytyqi, Gani Bajraktari, Michael Y. Henein

**Affiliations:** 1 Clinic of Cardiology, University Clinical Centre of Kosovo, 10000 Prishtina, Kosovo; 2 Institute of Public Health and Clinical Medicine, Umeå University, 90187 Umea, Sweden; 3 Riinvest College, 10000 Prishtina, Kosovo

**Keywords:** chronic coronary syndrome, percutaneous coronary intervention, optimal medial therapy

## Abstract

(1) Background and Aim: Conflicting evidence exists regarding the benefits of percutaneous coronary intervention (PCI) on survival and symptomatic relief of patients with chronic coronary syndrome (CCS) compared with optimal medical therapy (OMT). This meta-analysis is to evaluate the short- and long-term clinical benefit of PCI over and above OMT in CCS. (2) Methods: Main endpoints were major adverse cardiac events (MACEs), all-cause mortality, cardiovascular (CV) mortality, myocardial infarction (MI), urgent revascularization, stroke hospitalization, and quality of life (QoL). Clinical endpoints at very short (≤3 months), short- (<12 months), and long-term (≥ 12 months) follow-up were evaluated. (3) Results: Fifteen RCTs with a total of 16,443 patients with CCS (PCI *n* = 8307 and OMT *n* = 8136) were included in the meta-analysis. At mean follow-up of 27.7 months, the PCI group had similar risk of MACE (18.2 vs. 19.2 %; *p* < 0.32), all-cause mortality (7.09 vs. 7.88%; *p* = 0.56), CV mortality (8.74 vs. 9.87%; *p* = 0.30), MI (7.69 vs. 8.29%; *p* = 0.32), revascularization (11.2 vs. 18.3%; *p* = 0.08), stroke (2.18 vs. 1.41%; *p* = 0.10), and hospitalization for anginal symptoms (13.5 vs. 13.9%; *p* = 0.69) compared with OMT. These results were similar at short- and long-term follow-up. At the very short-term follow-up, PCI patients had greater improvement in the QoL including physical limitation, angina frequency, stability, and treatment satisfaction (*p* < 0.05 for all) but such benefits disappeared at the long-term follow-up. (4) Conclusions: PCI treatment of CCS does not provide any long-term clinical benefit compared with OMT. These results should have significant clinical implications in optimizing patient’s selection for PCI treatment.

## 1. Introduction

Coronary artery disease (CAD) is the commonest cardiovascular (CV) condition worldwide as well as the leading cause of morbidity and mortality and low quality of life. Chronic coronary syndrome (CCS) may have long stable periods but can also become unstable unexpectedly, although in most cases there is chronic disease progression [1]. The principal goals of treating patients with CCS are to alleviate symptoms, reduce the risk of major adverse cardiac events including death, and improve quality of life [2]. Guidelines recommend antianginal medications as the first line of treatment, with PCI performed for many patients who remain with persistent symptoms [3,4]. Many studies have shown that optimizing pharmacological therapy, lifestyle change, and education result in significant control of risk factors and consequently reduction in cardiovascular events [5,6,7]. Patients with moderate to severe symptoms and complex lesions who remain limited by angina, despite optimum medical therapy (OMT), are usually recommended for interventional treatment with the choice of the revascularization strategy determined by the coronary anatomy, individual’s comorbidities, and medical compliance [8,9]. Percutaneous coronary intervention (PCI) has advanced and its use has become increasingly crucial for accurately identified patients who are likely to experience symptom improvement. The European Society of Cardiology has provided the highest level of recommendation, Class I with Level of Evidence A, for using fractional flow reserve (FFR) to guide treatment decision making [10], in order to ascertain the anatomical and pathological causality of symptoms and the need for PCI as the best treatment. Despite the existing clear evidence for the benefit of early revascularization in reducing mortality and cardiac events in patients with ST elevation and non-ST elevation myocardial infarction [11,12], it is debatable whether PCI provides a prognostic advantage over and above OMT in the management of CCS. 

Unlike type 1 myocardial infarction for which the prompt revascularization is required, in CCS it has not been shown effective to reduce mortality or other cardiac events in the most patients compared with guideline-directed medical therapy [12]. Because the process of atherosclerosis is generally a systemic vascular and inflammatory condition affecting epicardial arteries and coronary microcirculation as well as other vascular beds, a comprehensive approach is necessary including intensive control of risk factors and pharmacological secondary prevention [13]. Moreover, while revascularization of epicardial coronary stenosis has been to provide better symptoms relief and to improve quality of life, particularly within a year after successful PCI, it is frequently associated with a need for subsequent coronary angiography to confirm/exclude an in-stent stenosis or residual coronary obstruction even if not significant. In these cases, residual angina is considered to be caused by non-obstructive disease that does not need PCI but OMT [13,14]. Thus, an often-unforeseen consequence of focusing disproportionately on epicardial coronary obstruction is that other pathogenetically important causes of angina and ischemia may not be considered. These causes include coronary microvascular dysfunction, microvascular coronary vasospasm, and derangements of myocardial energy or metabolism [14,15,16]. There is a need for a more inclusive management paradigm for patients with CCS to uncouple the association between obstructive CAD and revascularization. Data from recent large registries indicates that medical therapy can lead to improvement or resolution of self-reported angina in the majority of stable CAD patients, with revascularization being necessary only in a small percentage (5%) over the course of 5-year follow-up [16]. This requires identifying diagnostic and therapeutic approaches to better tailor treatment for both obstructive and non-obstructive causes of myocardial ischemia. By taking a pathogenetically directed approach, pharmacologic and procedural interventions can be aligned as complementary and synergistic for a broader population of CCS patients [14,17].

Undoubtedly PCI helps significantly in alleviating anginal symptoms of patients with CCS and obstructive lesions. Likewise, OMT does minimize, if not completely eradicate symptoms, in many of those patients. However, the respective outcome of the two treatment options remains uncertain, which should assist in better stratifying the patients for either of the two management options. Although PCI has become significantly safer, it carries a complication rate of 1–2% [18], likewise antianginal medications while beneficial can have side effects that prohibit their long-term use and quality of life [19].

The aim of this meta-analysis was to evaluate the short- and long-term benefit of PCI over and above OMT in CCS.

## 2. Methods

### 2.1. Search Strategy and Selection Criteria

We followed the PRISMA guidelines of the 2020 preferred reporting items for systematic reviews and meta-analysis statement [20]. Due to the study design (meta-analysis), neither Institutional Review Board (IRB) approval nor patient informed consent was needed. A PECOS model was used to develop the research question and search strategy, taking into account the population, exposure, comparison, outcomes, and study design (Table S1).

The following databases were searched from inception through to 10th July 2022: PubMed-Medline, EMBASE, Scopus, Google Scholar, the Cochrane Central Registry of Controlled Trials, and ClinicalTrial.gov, using the following key words: stable obstructive coronary artery disease, chronic coronary artery disease, chronic coronary syndrome, percutaneous coronary intervention, invasive strategy, optimal medical therapy, conservative strategy, outcome, mortality, revascularization, myocardial infarction, stroke, hospitalization, and quality of life (Table S2).

Additional searches for potential trials included reviewing the references of related review articles and the abstracts from the relevant congresses such as the scientific sessions of the European Society of Cardiology (ESC), European Atherosclerosis Society (EAS), the American Heart Association (AHA), American College of Cardiology (ACC), and European Association of Percutaneous Cardiovascular Interventions (EAPCI). The wild-card term ‘‘*’’ was used to enhance the sensitivity of the search strategy. The literature search was restricted to articles published in English and to human studies. No additional filters were applied. Two reviewers (DM and SB) independently and separately evaluated each article. Disagreements were resolved by discussion with the senior investigator (IB). The remaining articles were obtained in full-text and assessed by the same two researchers. For each trial, risk of bias was independently assessed by the same investigators using the revised Cochrane risk-of-bias tool for randomized trials (Cochrane RoB2 tool), involving five domains (randomisation process, deviation from intended interventions, missing outcome data, outcome measurement, and selection of reported results). The risk of bias in each study was judged to be “low”, “high” or “unclear” [21]. Articles were considered eligible if they reported the clinical outcome in patients with chronic coronary syndrome: (a) trials investigating CCS and reporting on the two arms (PCI and OMT); (b) randomized controlled trials; (c) follow-up trials; and (d) enrolled population of adults aged ≥18 years. Exclusion criteria were: (a) insufficient statistical data to compare two groups, (b) only one group of treatment, (c) patients with chronic total occlusion; (d) no follow-up, (e) studies not in humans, and (e) ongoing trials (unless they had reported relevant interim results). The following data were reviewed and extracted from eligible studies: (a) first author’s name; (b) year of publication; (c) name of the clinical trial; (d) country where the study was performed and number of centres; (e) study design; (f) number of participants in each studied group; (g) mean follow-up; (h) age and sex of study participants; (i) comorbidities; and (j) CV events. A total of 1241 articles were retrieved from the search after checking duplicates and were limited to randomized clinical trials in human, from the different databases. These articles were first screened by title and abstract, and the resulting 62 articles underwent full-text review. After a stringent selection process, 15 articles met the inclusion criteria. 

### 2.2. Outcome Variables

The primary endpoints were major adverse cardiac events (MACEs), all-cause mortality, cardiovascular mortality (CV), myocardial infarction (MI), urgent revascularization, stroke, and hospitalization for worsening angina. Secondary endpoint was quality of life (QoL). MACE was defined as: all-cause mortality, CV mortality, MI, revascularization, hospitalization for unstable angina and/or stroke. Quality of life was assessed based on Seattle Angina Questionnaire scores (SAQ scores), including physical limitation, angina frequency, angina stability, quality of life, and treatment satisfaction. The SAQ domain scores and the summary score, which range from 0 to 100, indicate the level of angina, functional limitations, and overall quality of life experienced by the patient. A higher score signifies less angina, fewer restrictions on daily activities, and an improved quality of life [22]. The effects of the treatment were evaluated over various time frames, including very short (up to 3 months), short-term (up to 12 months), and long-term (over 12 months) follow-up.

### 2.3. Data Synthesis and Statistical Analysis

The meta-analysis was conducted using Statistical analysis performed and the RevMan (Review Manager [RevMan] Version 5.1, The Cochrane Collaboration, Copenhagen, Denmark), with two-tailed *p* < 0.05 considered significant. Relative risk ratios (RRs) with 95% confidence interval (CI) are presented as summary statistics, and for continuous variable weighted mean differences (WMDs) the 95% CI was used. The baseline characteristics are reported in mean and standard deviation. Mean and standard deviation values were estimated using the method described by Hozo et al. [23]. Analysis is presented in forest plots, the standard way for illustrating the results of individual studies and meta-analysis. The Meta-analyses were performed with the random-effects model. Heterogeneity between studies was assessed using Cochrane Q test and I^2^ index. As a guide, I^2^ < 25% indicated low, 25–50% moderate and >50% high heterogeneity [24]. To assess the additive (between-study) component of variance, the reduced maximum likelihood method (*tau*^2^) incorporated the occurrence of residual heterogeneity into the analysis [25]. Based on the calculated value of risk ratio [RR]; when it is 1, above or below 1, we calculated the relative risk of CV events [26]. Publication bias was assessed using visual inspections of funnel plots and Egger’s test. 

## 3. Results

### 3.1. Study Selection and Patient Population

A total of 5714 articles were identified in the initial search, 1241 of which were screened as potentially relevant. After a stringent selection process, a total of 15 studies with 16,443 CCS patients (PCI *n* = 8307 and OMT *n* = 8136) and a mean follow-up of 27.7 months were included in the meta-analysis [27,28,29,30,31,32,33,34,35,36,37,38,39,40,41], Figure S1. 

Out of 15 articles, 10 RCTs with 9166 patients reported clinical events [27,28,30,31,32,34,35,36,37,40], 2 RCTs with 3697 patients reported quality of life [33,41] and three papers with 3580 patients reported both of them [29,38,39]. The characteristics of the included studies are shown in Table 1. The mean age of patients treated with PCI and OMT was: (65.4 ± 7.8 vs. 65.8 ± 7.9 years; *p* = 0.65), female gender distribution (33.0 vs. 34.3%; *p* = 0.48) and angina class more than class II (60.4 vs. 57.8%; *p* = 0.22) were not different between the two groups (Table S3).

### 3.2. Clinical Outcomes in the Two Treatment Groups

**Figure 1:**
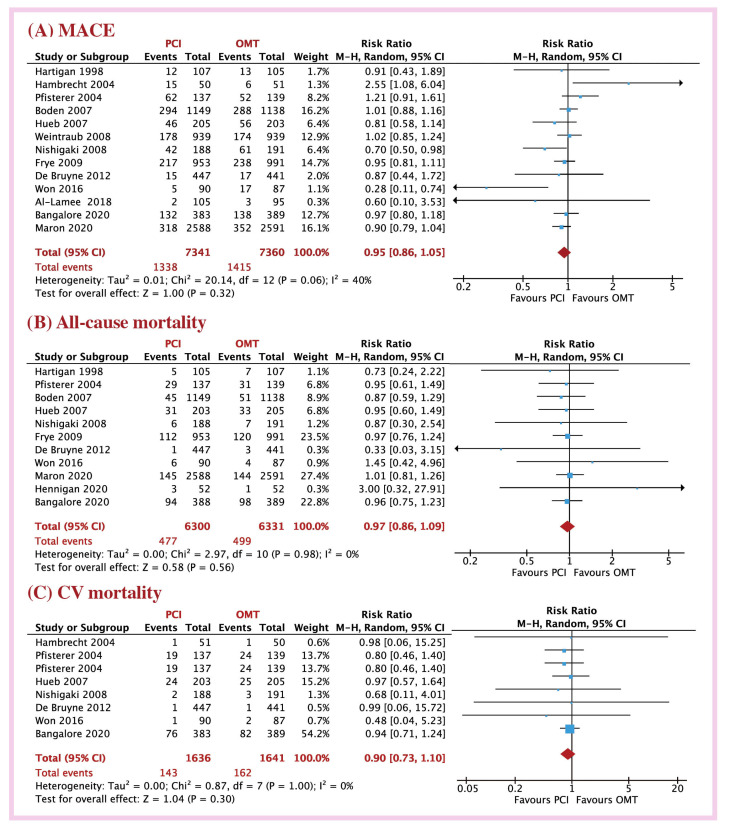
Risk ratio of outcome with PCI versus OMT in CCS patients; (**A**) MACE, (**B**) all-cause mortality, (**C**) CV mortality. Abbreviations: CCS: chronic coronary syndrome; PCI: percutaneous coronary intervention; OMT: optimal medical therapy; MACE: major adverse cardiac events; CV: cardiovascular.

**Figure 2:**
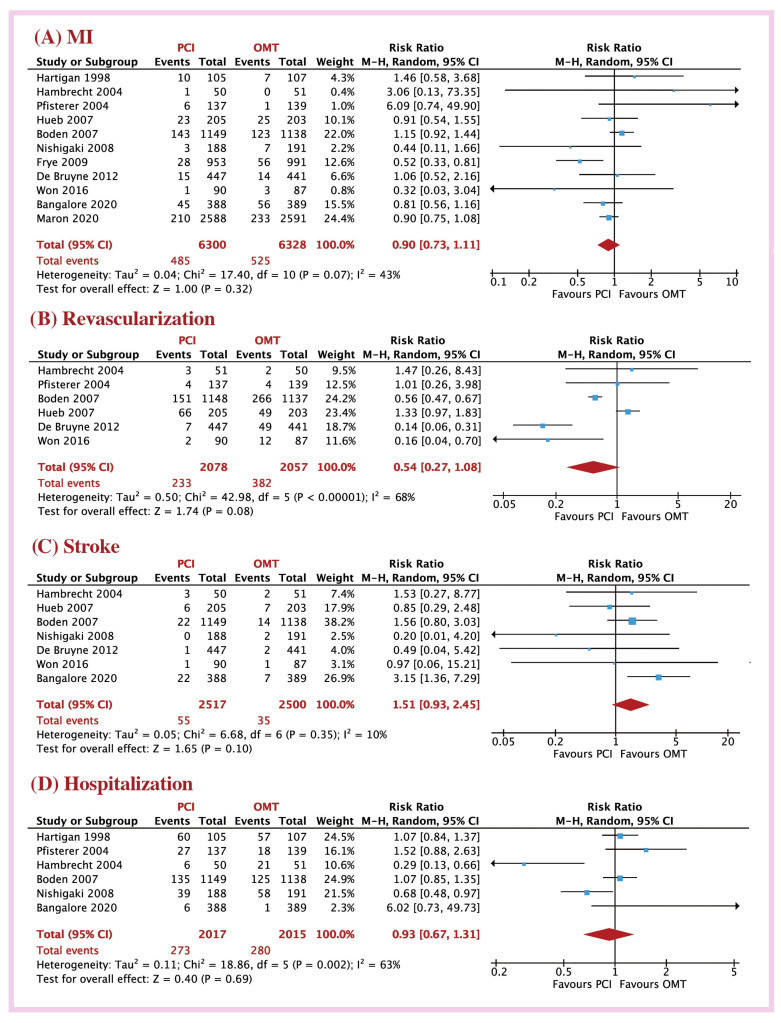
Risk ratio of outcome with PCI versus OMT in CCS patients; (**A**) MI, (**B**) revascularization, (**C**) hospitalization, (**D**) stroke. Abbreviations: CCS: chronic coronary syndrome; PCI: percutaneous coronary intervention; OMT: optimal medical therapy; MI: myocardial infarction.

At long-term follow-up (27.7 months), the PCI group had similar risk of MACE (18.8 vs. 19.6 %; RR = 0.97, 95% CI: 0.86 to 1.09, *p* < 0.56; I^2^ = 40%), all-cause mortality (7.09 vs. 7.88%; RR = 0.97, 95%CI: 0.86 to 1.09, *p* = 0.56; I^2^ = 0%), and CV mortality (8.74 vs. 9.87%; RR = 0.90, 95%CI: 0.73 to 1.10, *p* = 0.30; I^2^ = 40%; Figure 1) to OMT patients. Likewise, myocardial infarction (7.69 vs. 8.29%; RR = 0.90, 95%CI: 0.73 to 1.11, *p* = 0.32; I^2^ = 43%), revascularization (11.2 vs. 18.3%; RR = 0.54, 95%CI: 0.27 to 1.08, *p* = 0.08; I^2^ = 68%), stroke (2.18 vs. 1.41%; RR = 1.51, 95%CI: 0.93 to 2.45, *p* = 0.10; I^2^ = 10%), and hospitalization for symptoms (13.5 vs. 13.9%; RR = 0.93, 95%CI: 0.67 to 1.31, *p* = 0.69; I^2^ = 63%) were not different between the two treatment groups (Figure 2). These results were also not different between short- and long-term follow-up (*p* > 0.05 for all clinical events, Figures S2–S6). 

### 3.3. Quality of Life in the Two Treatment Groups

**Figure 3:**
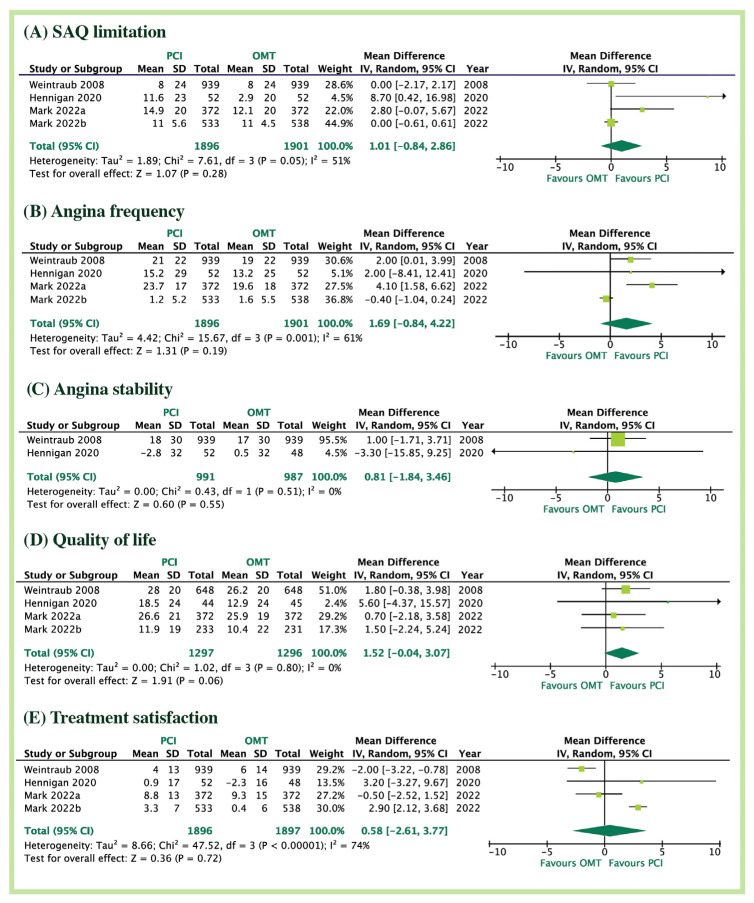
Quality of life with PCI versus OMT in long term; (**A**) physical limitation, (**B**) angina frequency, (**C**) angina stability, (**D**) quality of life, (**E**) treatment satisfaction. Abbreviations: PCI: percutaneous coronary intervention; OMT: optimal medical therapy; SAQ: Seattle Angina Questionnaire scores.

At long-term follow-up (12 months), the QoL analysis did not show any treatment-related differences: physical limitation (MD = 1.01, 95% CI: −0.84 to 2.86; *p* = 0.28; I^2^ = 51%), angina frequency, (MD = 1.69 95%CI: −0.84 to 4.22, *p* = 0.19; I^2^ = 61%), angina stability (MD = 0.81, 95%CI: −1.84 to 3.46, *p* = 0.55; I^2^ = 0%), quality of life score (MD = 1.52, 95%CI; −0.04 to 3.07, *p* = 0.07; I^2^ = 0%): and treatment satisfaction (MD = 0.58, 95%CI: −2.61 to 3.77, *p* = 0.72; I^2^ = 74%, Figure 3, Graphical abstract). Only at very short-term follow-up, patients treated with PCI had greater improvement in the QoL including physical limitation (MD = 0.12, 95% CI: 0.06 to 0.19; *p* = 0.003; I^2^ = 0%), angina frequency, (MD = 4.64 95%CI: 0.99 to 8.30, *p* = 0.01; I^2^ = 67%), angina stability (MD = 2.62, 95%CI: 0.16 to 5.08, *p* = 0.04; I^2^ = 0%), QoL (MD = 5.66, 95%CI; 2.30 to 8.82, *p* = 0.008; I^2^ = 72%), and treatment satisfaction (MD = 1.98, 95%CI: 0.06 to 3.90, *p* = 0.04; I^2^ = 72%, Figure S7). These differences disappeared at long-term follow-up.

### 3.4. Risk of Bias Assessment

The evaluation of the potential bias in the studies included in the analysis using RoB2 for RCTs revealed that a majority of the studies had a moderate to high level of quality in defining their objectives and the main outcomes (Table S4).

## 4. Discussion

Findings: The present meta-analysis of 15 trials with 16,443 CCS patients revealed the following: (a) PCI does not reduce the risk of MACE, all-cause mortality, CV mortality, MI, revascularization, stroke, or frequency of hospitalization for angina compared with OMT; (b) at the same long-term follow-up period, the two treatment strategies did not differ in their ability to impact symptoms; (c) finally, the short-term better angina control after PCI did not last for the long-term follow-up but required revascularisation.

Data interpretation: Atherosclerosis is a generalized vascular pathology which involves one or more arterial system, commonly the coronary and the carotid systems [42]. Although in the carotid system, the disease commonly affects both sides with somewhat various severity, it has different phenotypic pattern in the right and left coronary systems [43]. The commonest presentation of coronary atherosclerosis is plaque formation at the site of bifurcation, although not always exclusively, as plaques may also develop at mid-arterial segments. These atherosclerotic coronary manifestations are identified based on conventional angiographic examination [44,45]. Additional atherosclerosis manifestations in the segments that look normal on angiography can be detected by the presence of atherosclerotic plaques on CT examination [46]. Some of these patients may show extensive calcification but no single flow limiting lesion. The extensive calcification itself can be the cause of recurrent CCS through minimizing coronary flow reserve [47,48].

The two groups of patients we studied were a mean age 65 years, with no gender difference and all had confirmed CAD, suggesting the likelihood of classic diffuse coronary tree disease irrespective of the presence and severity of stenosis. Our results showed no difference in long-term clinical outcome, neither in hard endpoints nor even in symptoms, despite the short-term better symptomatic control in the PCI group. These findings support the diffuse nature of CAD in this age group of patients and highlight the beneficial response to OMT in stabilizing the disease. The results also strongly suggest that the mechanism of late clinical events is not necessarily a consequence of the ischemic vascular territory subtending a stenotic coronary segment but rather due to either the diffuse disease or the development of new plaque rupture in distal coronary segments, even in the absence of flow-limiting stenoses [49,50]. In addition, it is essential to take into account the coexisting comorbidities when managing CCS, as they are common among these patients and may contribute significantly to events. Recently the European registry reported the significant regional differences in CCS patients with respect to mortality, hospitalization rates, and cardiovascular control. The register highlighted a significant finding that merely controlling global risk factors proved inadequate. After one year, two-thirds of the patients diagnosed with CCS had LDL cholesterol levels higher than the recommended targets outlined in guidelines, with over one-third having a body mass index of 30 kg/m2 or higher. The majority of patients, 83.3%, had at least one of their parameters, such as LDL cholesterol, blood glucose, or blood pressure, not at the desired target level [51].

Our results support the need for PCI for better symptoms control in patients who are poorly managed solely by OMT [52]. In these patients, who likely have tight proximal lesions in a main artery with limited collateral circulation, would likely need additional mechanical crushing of the plaque in order to better control the ischemic implications [53,54], including symptoms and prognosis. These results are supported by the contemporary surgical results that showed no long-term clinical events benefit compared with OMT in patients with CCS [55]. Certainly, these results do not apply to acute coronary syndrome or patients with unstable angina [56,57], in whom the pathophysiology of symptoms and clinical presentation is quite different.

The benefit and mechanism of optimum medical therapy are very well-studied, including vasodilatation of healthy territories, collateral recruitment, and anti-inflammatory effect of statins. These benefits tend to stabilize the diffuse disease, particularly recently developed soft plaques [58,59,60]. Over the last decade the use of statin therapy for the treatment and prevention of coronary artery disease has been increasingly emphasized [61]. Moreover, we previously showed that although statins reduce LDL they result in long term increased vascular calcification but with less clinical events, hence the expected impact on stabilizing the inflammatory component of the arterial disease. Thus, in CCS, having no tight proximal lesions is likely to be associated with similar hard endpoints outcome irrespective of the treatment strategy, PCI or optimum medical therapy. Finally, optimal patient-centred outcomes can be achieved through a collaborative decision-making process involving the patient, family, and physician. Both invasive and conservative approaches may be appropriate according to individual cases and should be viewed as complementary strategies, rather than competing treatments, in CCS management.

Clinical implication: Our results provide strong evidence supporting the equal long-term benefit from OMT and PCI in patients with CCS. Although the former treatment is mechanical and the latter is medical, the similar long-term clinical outcome of the two strategies highlight the diffuse nature of coronary atherosclerosis as the main determinant of future events, as long as the disease is stable. These findings highlight the need for accurate stratification of patient’s presentation when selecting management options.

We propose that OMT should be always the first line of treatment for patients with CCS. PCI should be considered for patients with persistent symptoms and those with poorly tolerated OMT. In addition, FFR use in determining different severity of coronary lesions may be of help in achieving optimum patient stratification. Perhaps severe stenosis should benefit from PCI and moderate stenosis should be first tried on OMT, provided it is well-tolerated. Prognostic studies on this strategy should be designed in order to support/refute this proposal.

Limitations: Our analysis should be interpreted within the context of some limitations. Firstly, the information on severity of myocardial ischemia was not available for all studies, so complete disease match between the two treatment groups cannot be confirmed. Secondly, the analysed trials did not provide enough data on the disease duration or treatment duration, neither did they classify the patients on the basis of cardiac function, ventricular function, ejection fraction, etc. This limits any potential conclusions about the impact of medical treatment and extent of coronary disease as well as the impact of ejection fraction. We relied on the accuracy of patient’s enrolment in the trials we analysed in explaining our findings and deriving our conclusions.

Conclusions: In patients with CCS, the impact of PCI on long-term clinical outcome including mortality is not different from that resulting from optimum medical therapy, even in patients benefiting at short-term follow-up. These results should have significant clinical implications in optimizing patients’ selection for PCI treatment.

## Figures and Tables

**Table 1:** Main characteristics of trials included in the study.

Study (Trial) Year	Study Design	Location	Population	Sample Size (PCI/OMT)	Primary Endpoints	Other Endpoints	Follow-Up (Months)
Hartigan 1998	RCTs	USA	CCS	212	MACE, Death, MI		36
(VA study)	(double blinded)			(105/107)	Revascularization		
					Hospitalization		
Hambrecht 2004	RCTs	Germany	CCS	101	MACE, Death, MI		12
	(single blinded)			(50/51)	Revascularization		
					Hospitalization		
Pfisterer (TIME) 2004	RCTs,	Brasil	CCS	276	MACE, Death, MI	QoL	27
	(double blinded)			(137/139)	Revascularization		
					Hospitalization		
Hueb (MASS II) 2007	RCTs,	Switzerland	CCS	408	MACE, Death, MI		60
	(double blinded)			(205/203)	Revascularization		
					Hospitalization		
Boden 2007	RCTs,	USA	CCS	2287	MACE, Death, MI		55.2
	(double blinded)	Canada		(1149/1138)	Revascularization		
					Hospitalization		
Nishigaki (IMCJ)	RCTs,	Japan	CCS	284	MACE, Death, MI		4
2008	(double blinded)			(192/192)	Revascularization		
					Hospitalization		
Weintraub (COURAGE)	RCTs,	USA	CCS	1878		QoL	36
2008	(double blinded)	Canada		(939/939)			
Frye (BARI 2D) 2009	RCTs,	USA	CCS	1944	MACE, Death, MI		52.6
	(double blinded)			(953/991)			
De Bruyne (FAME 2)	RCTs	Europe	CCS	888	MACE, Death, MI		7.1
2012	(double blinded)	North USA		(447/441)	Revascularization		
					Hospitalization		
Won 2016	RCTs,	USA	CCS	177	MACE, Death, MI		12
	(double blinded)			(90/87)	Revascularization		
					Hospitalization		
Al-Lamee (ORBITA)	RCTs,	UK	CCS	200	MACE, Death, MI	QoL	1.5
2018	(double blinded)			(105/95)	Revascularization		
					Hospitalization		
Hennigan 2020	RCTs,	UK	CCS	104	Death	QoL	12
	(single blinded)			(52/52)			
Bangalore (ISCHEMIA	RCTs,	USA	CCS -CRF	777	MACE, Death, MI		26.2
-CKD) 2020	(double blinded)			(388/389)	Revascularization		
					Hospitalization		
Maron (ISCHEMIA)	RCTs,	USA	CCS	5179	MACE, Death, MI		38.4
2020	(double blinded)			(2588/2591)	Revascularization		
					Hospitalization		
Mark (ISCHEMIA)	RCTs,	USA	CCS	1819		QoL	36
2022	(double blinded)			(907/912)			

Abbreviations: CAD: coronary artery disease CCS: Chronic coronary syndrome; CRF: Chronic renal failure; CV: cardio-vascular; MI: myocardial infarction;. MACE: major adverse cardiac events; PCI: percutaneous coronary intervention; CABG: coronary artery bypass graft; QoL: quality of life; RCT: Randomised. Clinical Trial; NR: non-reported.

## Data Availability

Not applicable.

## Supplementary Materials

The following supporting information can be downloaded at: https://www.mdpi.com/article/10.3390/jcm12041395/s1, Figure S1: The Preferred Reporting Items for Systematic review and Meta-analysis (PRISMA) flow-chart of studies included in the meta-analysis; Figure S2: MACE in short- and long-term follow-up trials; Figure S3: All-cause mortality in short- and long-term follow-up; Figure S4: CV mortality in short- and long-term follow-up trials; Figure S5: MI in short- and long-term follow-up trials; Figure S6: Stroke in short- and long-term follow-up; Figure S7: Quality of life with PCI versus OMT in short term; Table S1: PECOS model; Table S2: Literature search strategy; Table S3: Main characteristics of patients enrolled among trials included in the study; Table S4: Assessment of risk of bias in the included studies using RoB2 for RCTs studies.
