# Supplementary material for: Percutaneous Coronary Intervention Is Not Superior to Optimal Medical Therapy in Chronic Coronary Syndrome: A Meta-Analysis
Source: J Clin Med. 2023 Feb 9;12(4):1395. doi: 10.3390/jcm12041395 (PMC9968177; doi:10.3390/jcm12041395)
Supplement: Supplementary file 1 [file jcm-12-01395-s001.zip › jcm-2132752-supplementary-afterproof_Final.pdf]

Journal of
Clinical Medicine

MDPI

SUPPLEMENTARY MATERIALS

Percutaneous Coronary Intervention Is Not Superior to Optimal Medical Therapy in Chronic Coronary Syndrome: A Meta-Analysis

Table S1. PECOS model.

PECOS Model	
Participants:	Patients with stable coronary artery disease
Exposure:	Trials investigating stable CAD, reporting in two arms: PCI and OMT
Comparator/Control:	PCI/OMT
Outcomes:	Primary outcomes: major adverse cardiac events (MACE), all-cause mortality, cardiovascular (CV) mortality, myocardial infarction (MI), urgent revascularization, stroke, and hospitalization.<br>Secondary outcome: quality of life.
Study design:	Previously systematic reviews or meta-analysis were not included but these articles were searched for individual trials

Table S2. Literature search strategy.

Database search	PubMed-Medline, EMBASE, Scopus, Google Scholar, the Cochrane Central Registry of<br>Controlled Trials, and ClinicalTrial.gov.
Statin intolerance term	" Stable obstructive coronary artery disease" OR "CCS" OR " Chronic coronary artery disease" OR "Percutaneous coronary intervention" OR "PCI" OR "Optimal medical therapy" OR "OMT" OR "Outcome" OR "Mortality" OR "Revascularization" OR "Myocardial infarction" OR "Stroke" OR "Hospitalization" OR "Quality of life" OR "QoL"
Trial type term	"Clinical outcome" OR "Mortality" OR "Myocardial infarction" OR "Revascularization" OR
	"Stroke" OR "Hospitalization" OR "Quality of life"
Additional search	European Society of Cardiology (ESC), the American Heart Association (AHA), European Atherosclerosis Society (EAS), American College of Cardiology (ACC), and European Association of<br>Percutaneous Cardiovascular Interventions (EAPCIs).

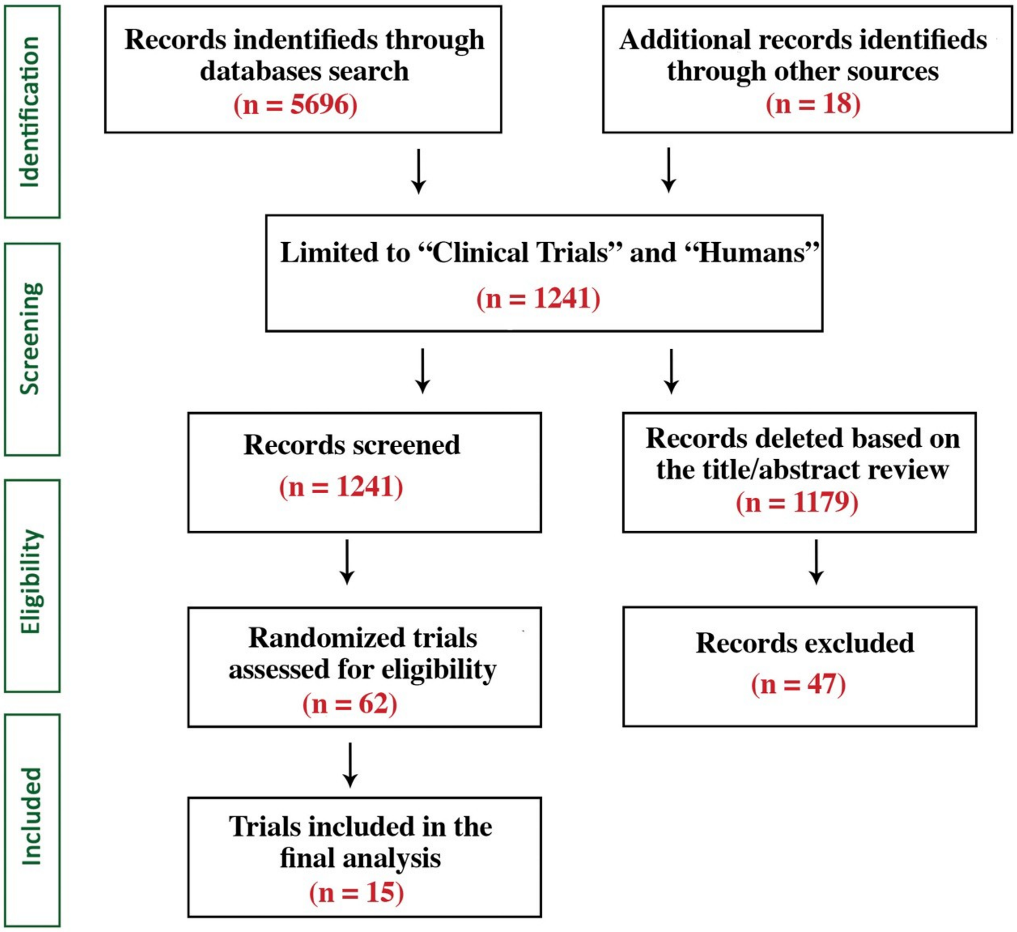

Figure S1. The Preferred Reporting Items for Systematic review and Meta-analysis (PRISMA) flow-chart of studies included in the meta-analysis.

Table S3. Main characteristics of patients enrolled among trials included in the study.

Study (Trial) Year	Groups	Age Year	Female %	HTN %	DM %	Dyslipidemia %	Smoking %	Angina CSS ≥ II	Prior MI	Prior PCI/CABG
Hartigan (VA study)	PCI	62	NR	52	17	NR	29	NR	33	NR
1998	OMT	63	NR	53	19	NR	33	NR	28	NR
Hambrecht 2004	PCI	60 ± 1	NR	70	22	86	16	70	39	65
	OMT	62 ±1	NR	82	23	77	18	59	52	50
Pfisterer (TIME) 2004	PCI	80 ± 4	42	48	20	50	37	100	42	18
	OMT	80 ± 4	42	49	22	40	32	100	49	20
Hueb (MASS II) 2007	PCI	60 ± 9	33	61	23	NR	27	78	28	18
	OMT	60 ± 9	31	55	36	NR	33	78	23	41
Boden 2007	PCI	61.5 ± 10	15	66	32	NR	44	58	38	27
	OMT	61.8 ± 9.7	15	67	35	NR	27	59	39	26
Nishigaki (IMCJ) 2008	PCI	64.5 ± 7.2	25	63.3	40.4	63.8	NR	52	14	10.1
	OMT	64.2 ±7.6	25	63.4	39.8	63.4	NR	42	14.1	7.1
Weintraub (COURAGE)	PCI	68 ± 10	23	86	37	NR	NR	32	NR	NR
2008	OMT	68 ± 10	22	78	48	NR	NR	31	NR	NR
Frye (BARI 2D) 2009	PCI	62.3 ± 8.8	29.6	NR	NR	NR	NR	10.2	31.7	22.9
	OMT	62.4 ± 9.0	29.7	NR	NR	NR	NR	7.2	32.4	24.2
De Bruyne (FAME 2)	PCI	63.5±9.3	20.4	77.8	27.5	73.8	19.9	45.6	37.1	17.9
2012	OMT	63.8 ±9.6	33.4	77.6	26.5	78.9	20.4	44.8	37.8	17.2
Won 2016	PCI	78.0±4.2	49	82.2	40	43.3	20	NR	6.7	NR
	OMT	78.3±4.7	52	74.7	32.2	44.8	16.1	NR	4.6	NR
Al-Lamee (ORBITA)	PCI	65.9 ±9	30	69	14	77	10	98	5.0	10
2018	OMT	66.1 ± 8	26	69	22	65	16	97	7.0	16
Henigan 2020	PCI	60 ± 8	23	19.2	73.1	40.3	40.3	NR	NR	NR
	OMT	61 ± 9	25	19.2	59.6	25	25	NR	NR	NR
Bangalore (ISCHEMIA-CKD) 2020	PCI	62 ± 10	30.9	58.2	NR	11.9	11.9	NR	16	22.7
	OMT	64 ±12	31.4	56	NR	9.8	9.8	NR	18.3	22.1
Maron (ISCHEMIA)	PCI	65.9 ± 9	23.4	50.6	77	56.7	56.7	NR	19.2	25.7
2020	OMT	66.1± 8	21.7	52	65	57.9	57.9	NR	19.2	22.9
Mark (ISCHEMIA) 2022	PCI	67 ±10	18.6	41.5	NR	NR	NR	NR	19.5	34.4
	OMT	67 ± 9	19.2	40.8	NR	NR	NR	NR	19.6	26.4

Abbreviations: HTN: hypertension; DM: diabetes mellitus; MI: myocardial infarction; PCI: percutaneous coronary intervention; CABG: coronary artery by-pass grafting; NR: non-reported. (*) whole group.

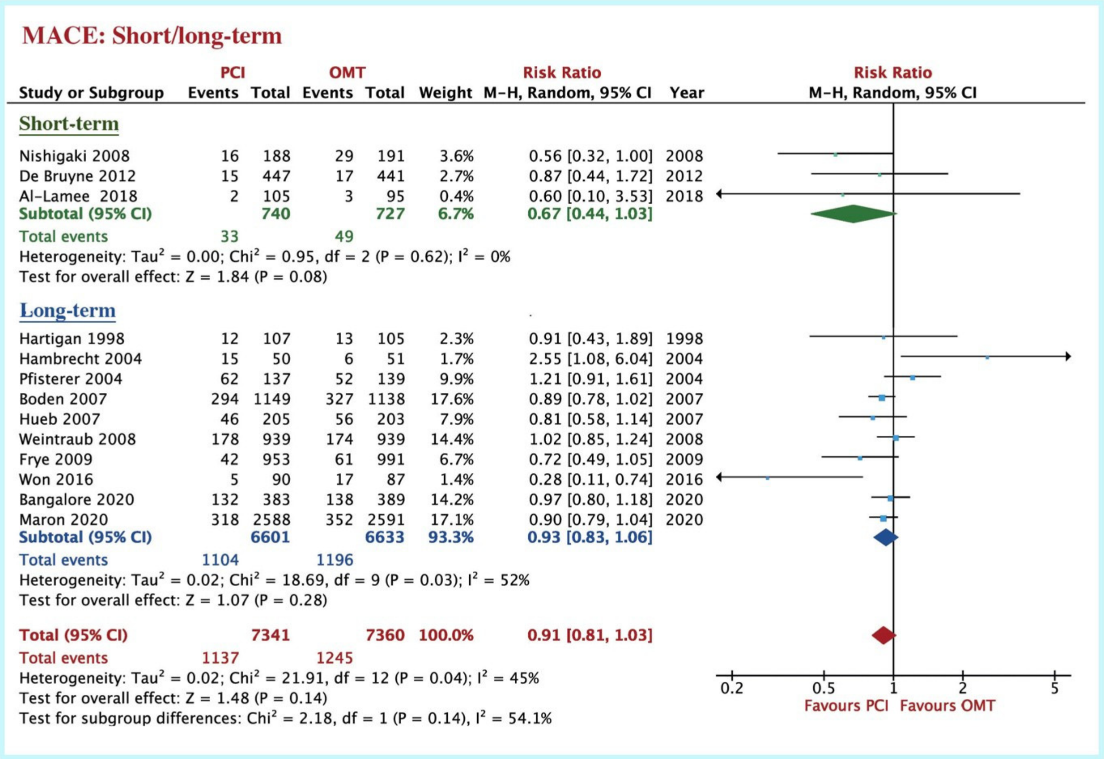

Figure S2. MACE in short- and long-term follow-up.

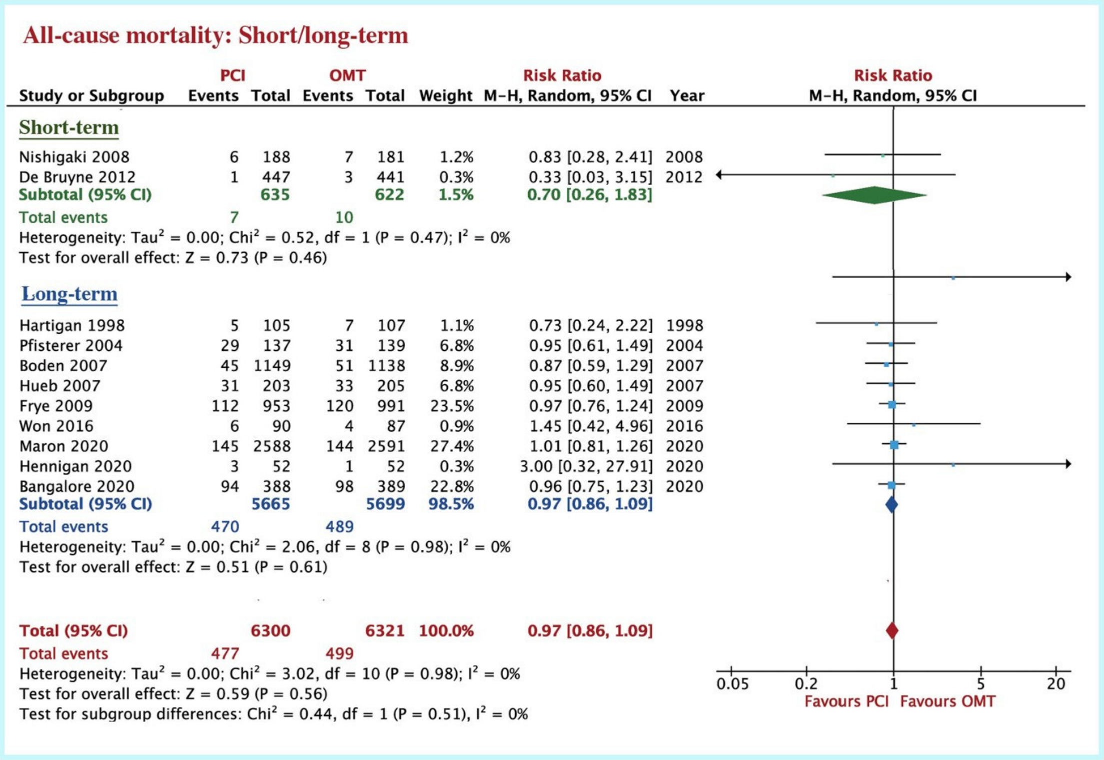

Figure S3. All-cause mortality in short- and long-term follow-up.

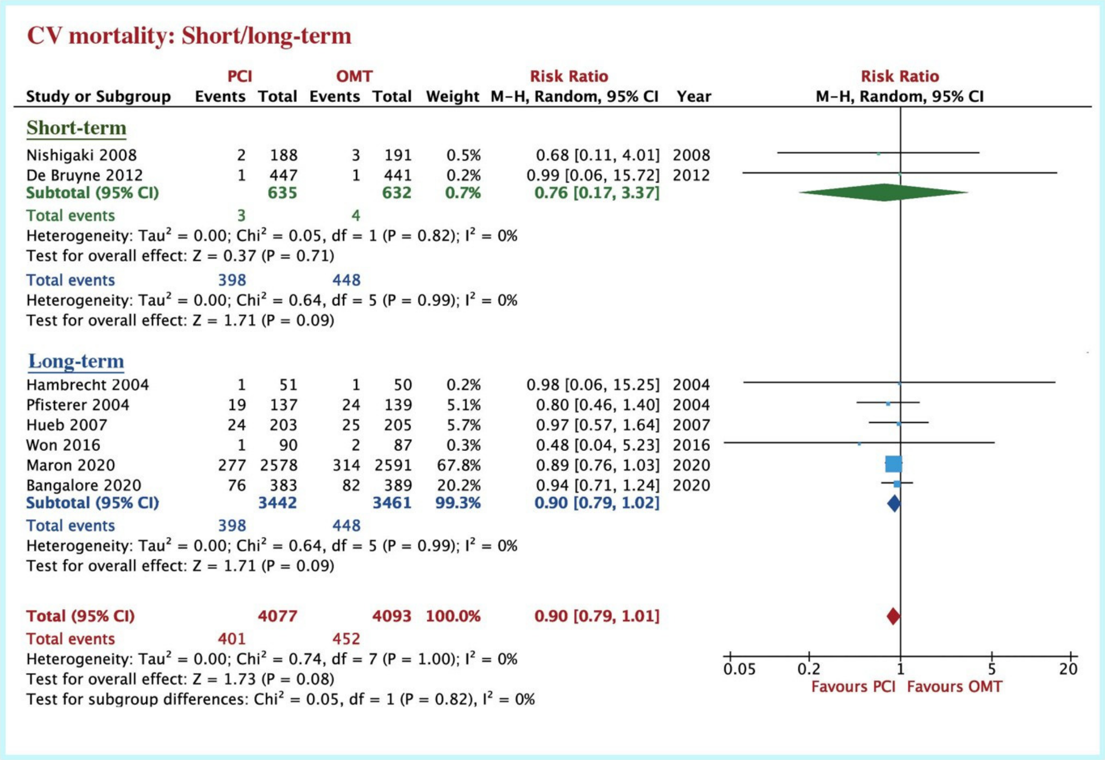

Figure S4. CV mortality in short- and long-term follow-up.

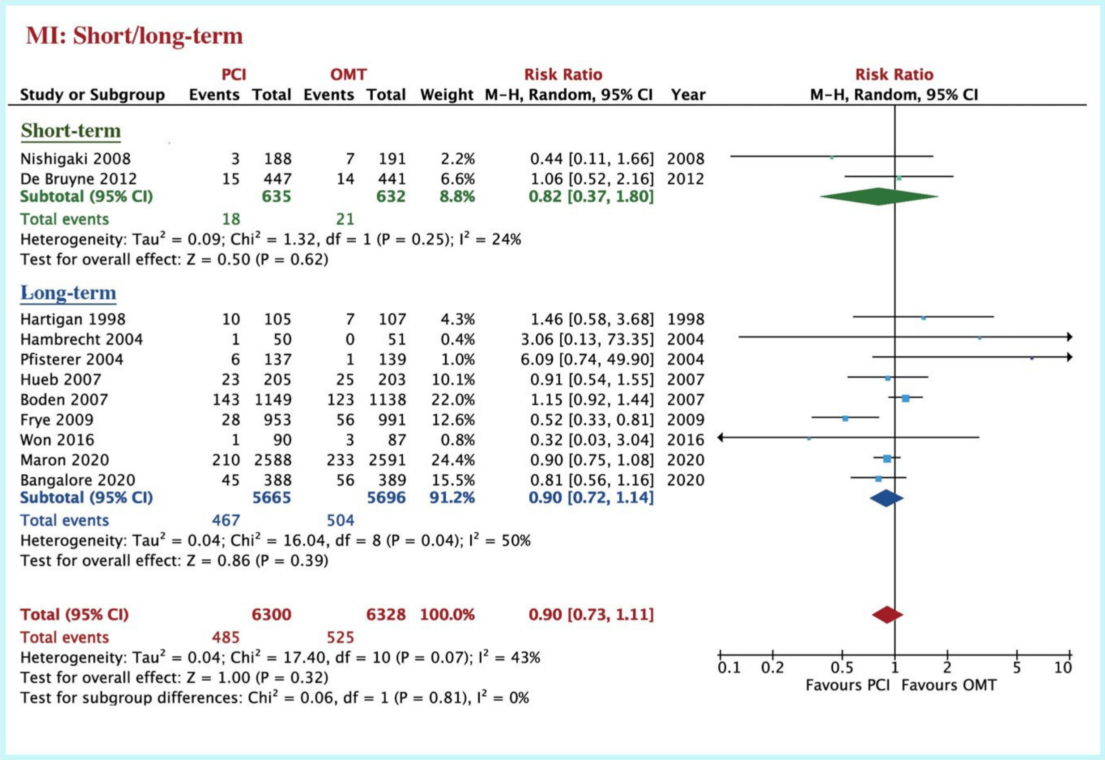

Figure S5. MI in short- and long-term follow-up.

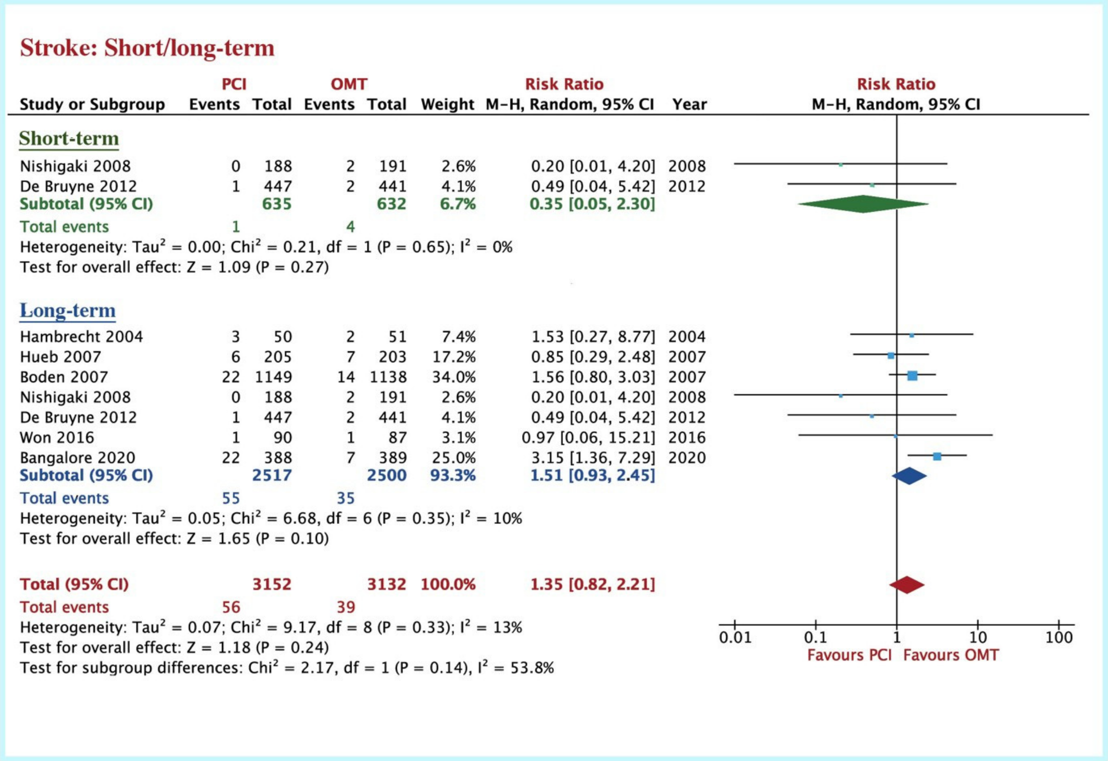

Figure S6. Stroke in short- and long-term follow-up.

(A) SAQ limitation

Study or Subgroup	PCI Mean	PCI SD	PCI Total	OMT Mean	OMT SD	OMT Total	Weight	Std. Mean Difference IV, Random, 95% CI	Year
Weintraub 2008	10	24	852	6	23	855	48.8%	0.17 [0.08, 0.27]	2008
Al-Lamee 2018	7.4	24	104	5	24	90	5.5%	0.10 [−0.18, 0.38]	2018
Hennigan 2020	16	26	52	11	23	48	2.8%	0.20 [−0.19, 0.59]	2020
Mark 2022a	12.5	22	307	10.2	22	303	17.5%	0.10 [−0.05, 0.26]	2022
Mark 2022b	4.4	19	439	3.7	16	446	25.4%	0.04 [−0.09, 0.17]	2022
**Total (95% CI)**			**1754**			**1742**	**100.0%**	**0.12 [0.06, 0.19]**	

Heterogeneity: $Tau^2 = 0.00$; $Chi^2 = 2.71$, df = 4 (P = 0.61); $I^2 = 0\%$
Test for overall effect: Z = 3.62 (P = 0.0003)

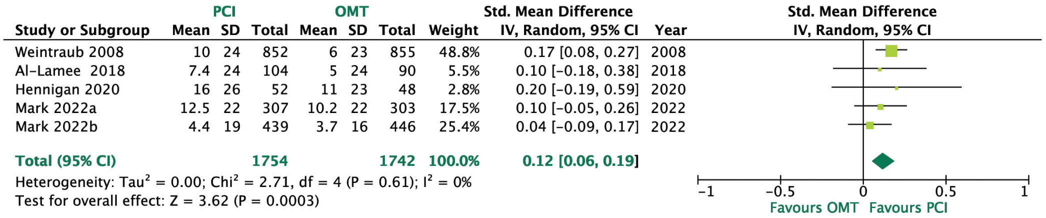

(B) Angina frequency

Study or Subgroup	PCI Mean	PCI SD	PCI Total	OMT Mean	OMT SD	OMT Total	Weight	Mean Difference IV, Random, 95% CI	Year
Weintraub 2008	17	22	871	11	23	873	25.4%	6.00 [3.89, 8.11]	2008
Al-Lamee 2018	11.2	22	104	7.4	22	90	15.4%	3.80 [−2.41, 10.01]	2018
Hennigan 2020	21	28	47	10	24	52	8.6%	11.00 [0.67, 21.33]	2020
Mark 2022a	2.5	12.2	505	2.2	10.3	505	26.7%	0.30 [−1.09, 1.69]	2022
Mark 2022b	21.4	18	354	15.1	20	353	23.9%	6.30 [3.49, 9.11]	2022
**Total (95% CI)**			**1881**			**1873**	**100.0%**	**4.64 [0.99, 8.30]**	

Heterogeneity: $Tau^2 = 12.55$; $Chi^2 = 29.70$, df = 4 (P < 0.00001); $I^2 = 67\%$
Test for overall effect: Z = 2.49 (P = 0.01)

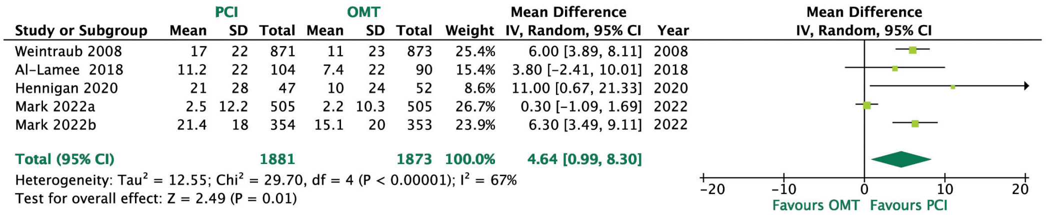

(C) Angina stability

Study or Subgroup	PCI Mean	PCI SD	PCI Total	OMT Mean	OMT SD	OMT Total	Weight	Mean Difference IV, Random, 95% CI	Year
Weintraub 2008	23	28	860	20	27	860	89.4%	3.00 [0.40, 5.60]	2008
Al-Lamee 2018	4.2	33	102	5.1	32	89	7.1%	−0.90 [−10.13, 8.33]	2018
Hennigan 2020	4	34	48	4	33	52	3.5%	0.00 [−13.15, 13.15]	2020
**Total (95% CI)**			**1010**			**1001**	**100.0%**	**2.62 [0.16, 5.08]**	

Heterogeneity: $Tau^2 = 0.00$; $Chi^2 = 0.79$, df = 2 (P = 0.67); $I^2 = 0\%$
Test for overall effect: Z = 2.09 (P = 0.04)

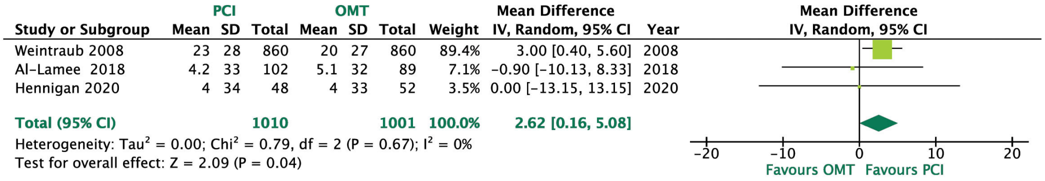

(D) Quality of life

Study or Subgroup	PCI Mean	PCI SD	PCI Total	OMT Mean	OMT SD	OMT Total	Weight	Mean Difference IV, Random, 95% CI	Year
Weintraub 2008	22	22	873	17	23	882	32.2%	5.00 [2.89, 7.11]	2008
Hennigan 2020	24	26	52	11	24	48	8.5%	13.00 [3.20, 22.80]	2020
Mark 2022a	22.6	22	352	14.5	22	351	27.1%	8.10 [4.85, 11.35]	2022
Mark 2022b	6.1	18	498	4.1	16	496	32.1%	2.00 [−0.12, 4.12]	2022
**Total (95% CI)**			**1775**			**1777**	**100.0%**	**5.56 [2.30, 8.82]**	

Heterogeneity: $Tau^2 = 7.45$; $Chi^2 = 13.17$, df = 3 (P = 0.004); $I^2 = 77\%$
Test for overall effect: Z = 3.34 (P = 0.0008)

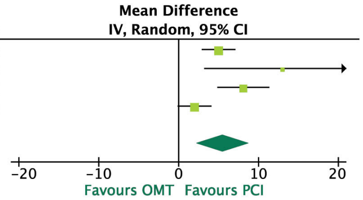

(E) Treatment satisfaction

Study or Subgroup	PCI Mean	PCI SD	PCI Total	OMT Mean	OMT SD	OMT Total	Weight	Mean Difference IV, Random, 95% CI	Year
Weintraub 2008	4	12	869	4	14	873	34.5%	0.00 [−1.22, 1.22]	2008
Hennigan 2020	2	13	52	−4	20	48	6.9%	6.00 [−0.67, 12.67]	2020
Mark 2022a	8	13	333	5	15	329	26.8%	3.00 [0.86, 5.14]	2022
Mark 2022b	2.1	13	471	−0.3	11	479	31.9%	2.40 [0.87, 3.93]	2022
**Total (95% CI)**			**1725**			**1729**	**100.0%**	**1.98 [0.06, 3.90]**	

Heterogeneity: $Tau^2 = 2.40$; $Chi^2 = 10.63$, df = 3 (P = 0.01); $I^2 = 72\%$
Test for overall effect: Z = 2.02 (P = 0.04)

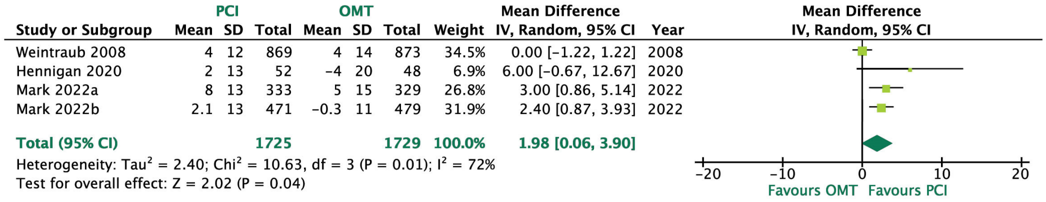

Figure S7. Quality of life with PCI versus OMT in short term. (**A**) Physical limitation, (**B**) angina frequency, (**C**) angina stability, (**D**) quality of life, (**E**) treatment satisfaction.

Table S4. Assessment of risk of bias in the included studies using RoB2 for RCTs studies.

Study, year	Randomization process	Deviation from intervention	Missing outcome data	Measuremen of the outcome	Selection of the results	Overall
Hartigan (VAstudy) 1998	L	L	S	L	L	S
Hambrecht 2004	L	L	S	L	S	S
Pfisterer (TIME) 2004	L	L	L	L	L	L
Hueb (MASS II) 2007	L	L	L	L	L	L
Boden 2007	L	L	L	L	L	L
Nishigaki (IMCJ) 2008	L	L	L	L	L	L
Weintraub	L	L	L	L	L	L
Frye (BARI 2D) 2009	L	L	L	L	S	S
De Bruyne (FAME 2)2012	L	L	L	L	L	L
Won 2016	L	L	L	L	L	L
Al-Lamee (ORBITA) 2018	L	L	L	L	L	L
Henigan 2020	L	L	L	L	L	L
Bangalore (ISCHEMIA-CKD)	L	L	L	L	L	L
Maron (ISCHEMIA) 2020	L	L	L	L	L	L
Mark (ISCHEMIA) 2022	L	L	L	L	L	L

L: low; **S:** some concerns; **H:** high.

Overall risk-of-bias judgement	Criteria
Low risk of bias	The study is judged to be at low risk of bias for all domains for this result.
Some concerns	The study is judged to raise some concerns in at least one domain for this result, but not to be at high risk of bias for any domain.
High risk of bias	The study is judged to be at high risk of bias in at least one domain for this result. The study is judged to have some concerns for multiple domains in a way that substantially lowers confidence in the result.
